# Critical Pertussis in a Young Infant Requiring Mechanical Ventilation

**DOI:** 10.1155/2013/125043

**Published:** 2013-05-02

**Authors:** Heda Melinda Nataprawira, Dadang Hudaya Somasetia, Sri Sudarwati, Minerva Kadir, Nanan Sekarwana

**Affiliations:** Department of Child Health, Faculty of Medicine, Universitas Padjadjaran, Hasan Sadikin General Hospital, Pasteur 38, Bandung, West Java 40161, Indonesia

## Abstract

Pertussis may likely be misdiagnosed in its initial or catarrhal phase as a common respiratory infection. The earlier diagnosis of pertussis really depends on the capability of the medical professional especially in the first line public health services. The lack of awareness in diagnosis of severe pertussis as one of the causes of severe respiratory problems may likely misdiagnose pertussis as respiratory failure or even septic shock. In fact, pertussis may manifest as a critical pertussis which can be fatal due to the respiratory failure that require pediatric intensive care unit using mechanical ventilation. We reported a confirmed pertussis case of a 7-weeks-old female infant referred to our tertiary hospital with gasping leading to respiratory failure and septic shock requiring mechanical ventilation, aggressive fluid therapy, and antibiotics. Pertussis was diagnosed late during the course of illness when the patient was hospitalized. Improvement was noted after administering macrolide which gave a good response. *Bordetella pertussis* isolation from Bordet-Gengou media culture yielded positive result.

## 1. Introduction

Pertussis can affect infant, children, and adolescence, but mostly children younger than 10 years [1]. In 2004, 35 percent cases occurred in the 10–14 year age group and only 18 percent cases in infants which reported an increase in infants group in 2005 [2]. 

Manifestation in infants is usually catastrophic. Severe pertussis leads to critical pertussis may manifest fatal for infants under three months of life because the symptoms may present themselves as other causes of illness such as sepsis, very severe pneumonia, and encephalopathy, which may result in respiratory and cardiovascular disturbances [3]. 

In reality, infection due to *Bordetella pertussis (B. pertussis)* can mimic other respiratory pathogens infection such as *respiratory syncytial virus* (RSV), adenovirus, rhinovirus, parainfluenza virus, *Mycoplasma pneumonia,* and *Chlamydia pneumonia,* so it is nearly impossible to distinguish them without microbiological confirmation [4]. This happens due to the nonspecific symptoms in early catarrhal phase and/or doctor's unawareness of pertussis diagnosis. It was reported that all pertussis cases that were diagnosed in Hasan Sadikin General Hospital during 2008–2010 were first diagnosed as severe bacterial pneumonia [5]. A child with severe probable pertussis may require care in the intensive care unit when apnea, very severe pneumonia, or respiratory failure along with circulation disturbance occurs. In the United States, the morbidity caused by pertussis in the pediatric intensive care unit was reported in about 1.5−8% [6]. The severity of pertussis, and the rapidity of its progression in young infants are affected by a number of factors such as the presence of transplacentally acquired maternal antibodies to *B. pertussis*, the infectious dose of bacteria that the infant receives, coinfection with respiratory viruses and perhaps genetic factors related to the pathogen or the infant, the source of pertussis which is usually is a household contact (most often the mother), and immunization status [7]. A confirmed pertussis diagnosis is so difficult which leads World Health Organization (WHO) and Center of Disease Control and Prevention (CDC) to define pertussis cases as probable and confirmed [8, 9]. Lower result of Bordet-Gengou media for *B. pertussis* isolation was reported with decreased sensitivity and increased false negative after the first two weeks [10]. We illustrated a case of a 7-week-old female infant without the history of diphtheria-pertussis-tetanus (DPT) vaccination who presented with respiratory failure and circulation disturbance and finally treated successfully.

## 2. Case Report

**Figure 1:**
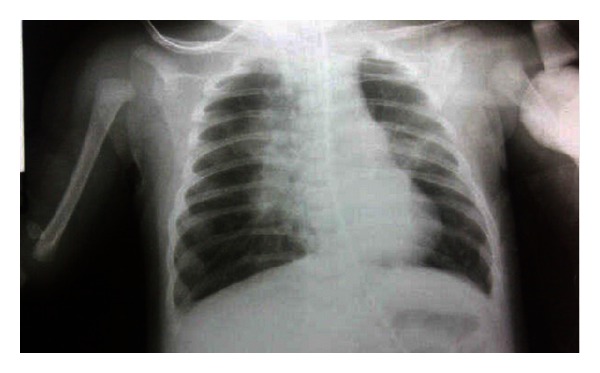
Anteroposterior thorax imaging showed bilateral pneumonia and no cardiac enlargement.

A 7-week-old female infant was admitted to our hospital with gasping and mottling along with perioral cyanosis at presentation. These symptoms which have been associated with coughing and fever 8 days before admission led parents to bring her to public health medical service who gave antibiotics and symptomatic medication. There was no history of postcough vomiting or breath cessation before. Since there was no clinical improvement and the patient's condition was worsening, she was referred to our tertiary hospital. She had no history of diphtheria-pertussis-tetanus (DPT) vaccination. She came from a low social economic family and nonsupporting environment with poor ventilation. On admission, she looked severely ill with fever (40°C), gasping, and bounding tachycardia with O_2_ saturation decreased to 84%. Physical examination revealed perioral cyanosis, nasal flaring, chest retraction, and crackles in both lung on auscultation. Abdominal findings were normal. Mechanical ventilation was set, and she got aggressive fluid therapy, cephalosporin antibiotic, and dopamine as well. Blood test result showed anemia (9.7 g/dL), leukocytosis (68,000/mm^3^) with absolute lymphocytosis (54%), and thrombocytosis (690,000/mm^3^). Blood smear result showed toxic granules and hypersegmentation. Blood gas analysis result showed metabolic acidosis and hypoxemia. Suggested bilateral pneumonia was noted on chest radiograph (Figure 1). Severe bronchopneumonia with respiratory and cardiovascular failure (septic shock) and anemia due to underlying disease was diagnosed. She was on ventilator for 5 days. On the 7th day of hospitalization, whooping cough was noted, and macrolide was administered. Nasopharyngeal swab for *B. pertussis* isolation in Bordet-Gengou media was taken, and it yielded positive result. Severe probable pertussis was then diagnosed. We conducted echocardiography to detect the probable pulmonary hypertension resulting in no pulmonary hypertension and good ventricular function. She was placed in isolation ward at the 9th day of hospitalization, showing clinical improvement and being discharged with better condition.

## 3. Discussions 

From further medical history, we found that this patient had been treated in peripheral health care facilities for twice and treated acute respiratory infection and then further referred to our tertiary hospital as a severe pneumonia. In the emergency department, she presented with respiratory failure along with vascular disturbance and she was given emergence fluid therapy, mechanical ventilation and antibiotics as well. Initially she was not diagnosed with pertussis as a cause of her condition. According to CDC, classic pertussis can be defined into three phases such as catarrhal phase with rhinorrhea with unspecific cough as symptoms, paroxysmal phase showing increasing severity of cough and coughing spells, frequently followed by whooping and post-cough vomiting, and convalescent phase [10]. In this final phase, the frequency and severity of cough will decrease. In this patient, the symptoms led to misdiagnosis of pertussis infection, and in fact, she further presented in advanced severe acute respiratory infection disease, which was severe pertussis. The definition of severe pertussis itself is still unclear although it can be classified as more severe and less severe [9]. Paroxysmal phase often ends in respiratory failure, apnea, seizure, and cardiogenic circulation disturbance. Our critical ill patient admitted to our hospital in paroxysmal phase needed intensive care unit and it is called is critical pertussis [3]. In Germany, it was reported that 12 of 234 babies died because of sudden infant death syndrome (SIDS) caused by *B. pertussis* [11]. 

Actually, we could diagnose probable pertussis on the basis of clinical presentation such as cough illness for more than two weeks with at least one of the following condition like paroxysms, whooping cough, and postcough vomiting without other apparent causes reported by health professional [8]. The probable pertussis can be guided by laboratory findings such as leukocytosis and absolute lymphocytosis. Leukocytosis is found in catarrhal phase estimated 15,000–100,000/mm^3^. The severity of leukocytosis is associated with poor outcome of *B. pertussis* infection. It is suggested that the toxins produced by *B. pertussis* induced lymphocyte proliferation and activation and elevated the cAMP levels which contribute to pulmonary vasoconstriction [12]. Leukocytes aggregate within the pulmonary circulation and form a mechanical obstruction to transpulmonary blood flow with the result being severe hypoxemia and pulmonary hypertension. In our case, echocardiography examination did not identify pulmonary hypertension. It was reported in Chile from autopsy that three out of five pertussis death cases were due to pulmonary hypertension [13]. Culture is the gold standard to define pertussis and Bordet-Gengou is a choice for routine. However, positive culture of *B. pertussis* is difficult to be obtained as the test may be affected by specimen collection, transportation, and isolation techniques. Higher isolation result is obtained during the catarrhal and early paroxysmal stages, two weeks during course of illness, with its positive variation of 30%–50% [14]. We found positive Bordet-Gengou result in this patient. In this case, severe leukocytosis might likely as a cause of respiratory failure. This critical pertussis caused hypotension and shock that mimicked severe sepsis. In critical pertussis, intensive care unit is required and several cases require assistance of ventilation, especially in infants with apnea, severe pneumonia and respiratory failure [3]. Treatment of pertussis should include the management of symptomatic signs and direct to the causative organism with macrolide antibiotic. Symptomatic treatment aims at decreasing coughing and support nutrition. Erythromycin is the traditional treatment, however, multicentered randomized controlled trials in children reported that the newer macrolides azithromycin and clarithromycin have similar effectiveness, better compliance with less side effects compared with erythromycin [15, 16]. Dosage uses in treatment erithromycin can be 40–50 mg/kg of body weight/day (maximum two g per day) in four divided doses for 14 days. Clarithromycin dosage is 15 mg/kg of body weight/day (maximum one g per day) in two divided doses each day for 7 days. Azithromycin was given 10 mg/kg of body weight/day for five days [16, 17]. 

Immunization can reduce the severity of breakthrough disease in children who receive three doses of vaccine compared with that in unvaccinated children. In our case, the patient has not received DPT vaccination as her age was under 2 months of age when suffering from the disease. So, it is likely that she got the infection from adults surrounding her. The predominant factors for this patient were no history of immunization, low social economy, and nonsupporting household environment [18].

## 4. Conclusions

Pertussis can be presented in fatal respiratory failure if it is diagnosed late and not treated appropriately. The clinical presentation is often difficult to observe by parents and peripheral medical services, and it causes delay in diagnosis and treatment. It is noteworthy that we concern about probable pertussis in young infants with long-lasting cough particularly associated with postcough vomiting and/or perioral cyanosis, having no DPT immunization, apnea, or whooping cough, and for this disease, macrolides are drug of choice.
